# A modeling approach to compute modification of net joint forces caused by coping movements in obstetric brachial plexus palsy

**DOI:** 10.1186/1749-7221-8-10

**Published:** 2013-10-21

**Authors:** Tim Kleiber, Nikica Popovic, Jörg Bahm, Catherine Disselhorst-Klug

**Affiliations:** 1 Department of Rehabilitation and Prevention Engineering, Institute of Applied Medical Engineering, RWTH Aachen University, Aachen, Germany; 2 German Research School for Simulation Sciences, Jülich, Germany; 3 Department of Reconstructive Surgery, Franziskus Hospital, Aachen, Germany

**Keywords:** Biomechanics, Kinematics, Kinetics, Internal shoulder rotation, Inverse dynamic, Joint malformation

## Abstract

**Background:**

Many disorders of the musculoskeletal system are caused by modified net joint forces resulting from individual coping movement strategies of patients suffering from neuromuscular diseases. Purpose of this work is to introduce a personalized biomechanical model which allows the calculation of individual net joint forces via inverse dynamics based on anthropometry and kinematics of the upper extremity measured by 3D optoelectronical motion analysis.

**Methods:**

The determined resulting net joint forces in the anatomical axis of movement may be used to explain the reason for possible malfunction of the musculoskeletal system, especially joint malformation. For example the resulting net joint forces in the humerothoracic joint from simulations are compared to a sample of children presenting obstetric brachial plexus palsy showing an internal shoulder rotation position and a sample of healthy children.

**Results:**

The results presented from the simulation show that an increased internal shoulder rotation position leads to increased net joint forces in the humerothoracic joint. A similar behavior is presented for the subjects suffering from brachial plexus palsy with an internal shoulder rotation position.

**Conclusions:**

The increased net joint forces are a possible reason for joint malformation in the humerothoracic joint caused by coping movements resulting from neuromuscular dysfunction as stated in literature.

## Background

Based on the increasing number of people affected by orthopedic disorders, maximum support in early diagnosis is necessary to guarantee fast intervention and recovery. Neuromuscular dysfunction may lead to muscular imbalance where the missing antagonistic muscle force caused by a palsy may force a patient to perform pathological coping movements. These coping movements may have a large impact on movement strategies and consequently on the magnitude and direction of the acting net joint forces which may increase the risk of joint malformation [1,2]. To calculate and minimize this risk, the individual movement strategies of every patient and their specific dysfunction must be analyzed and taken into account for therapy planning.

Apart from visual observation by clinicians, 3D motion analysis is a useful technique and an established tool for the diagnosis of movement disorders and the quantitative evaluation of human movement. Today the standardized measurement of motion analysis is common in clinical gait analysis [3,4]. In the last years the development of rigid body models for the upper extremity were also established, but the investigation of captured motion is limited by the availability of suitable biomechanical models [5-7].

For the kinetic description of motion it is necessary to measure the forces acting on the body during movement. In gait analysis these external loadings are easily acquired using force plates which detect the ground reaction forces and moments [8]. Gait parameters in healthy subjects are cyclic and easy to reconstruct, in the upper extremity it is more difficult because of the complexity and the degrees of freedom in arm movements. Upper extremity movements are inter-individually different, unconstrained and non-cyclic. Standardizing upper extremity movements is extremely complex in contrast to gait. One approach to solve these problems is an end-effector based force/torque sensor installed on a robot arm to standardize predefined movements and record the external forces synchronously [9].

In addition the acquired kinematics of movement can be used in conjunction with anthropometric data as input to determine net joint forces of the whole joint chain by inverse dynamics [9]. This individual mechanical joint loading during movement is considered to have the main impact on joint malformation in upper extremity resulting from subject specific coping movements [1,2,10].

The purpose of this study is to show a possible impact of increased net joint forces resulting from coping movements on malformation in the musculoskeletal system. This is shown by the application of a personalized biomechanical inverse dynamic model. As an example, we focus on joint malformation of the humerothoracic joint as it relates to an internal shoulder rotation position during shoulder flexion and extension movements. This malformation finds its origin by the presence of increased pathological net joint forces during shoulder flexion and extension movements, resulting from an internal shoulder rotation position presented in a specific patient group suffering from obstetric brachial plexus palsy.

## Methods

### Subjects

Subjects suffering from obstetric brachial plexus palsy are often using coping movements in everyday situations when they are restricted in their standard movements through muscular imbalance. In literature especially in these patients a malposition in the humerothoracic joint has been reported. Most common malposition that occurs within brachial plexus palsy is a shortening of the internal rotators due to the palsy of the antagonistic muscle group in the shoulder and so a resulting internal shoulder rotation position [1,2,11,12].

For this study, as an example, a group of four children presenting obstetric brachial plexus palsy with an internal shoulder rotation position was investigated and compared to a sample of healthy children. The affected children are all male, in the age between 8 and 11 years and all suffering from obstetric brachial plexus palsy. They are all affected by an internal rotation position of the shoulder joint reaching from 20° to 40° in standing position. They all have a similar, non-restricted range of motion in shoulder flexion and did not have any surgical intervention before. The control group consists of four male children in a similar age and motor development.

All actions during the measurement procedure were performed according to the Declaration of Helsinki and all subjects and/or their parents/guardians/next of kin were informed about the experimental protocol and the potential risks of the study and gave written informed consent for the publication of this paper and pictures taken during measurements before their participation.

### Determination of body segment parameters

To personalize a biomechanical model the subject’s anthropometric data is required in order to determine body segment parameters such as segment mass, segment’s center of mass and segment’s moment of inertia. Using the linear regression equations of Zastiorsky and Seluyanov [13], all body segment parameters can be estimated and scaled by the subject’s weight and height as well as the segments length. These body segment parameters are used as input in conjunction with the kinematics of movement to calculate the net joint forces of the whole joint chain.

### Determination of kinematics

**Figure 1:**
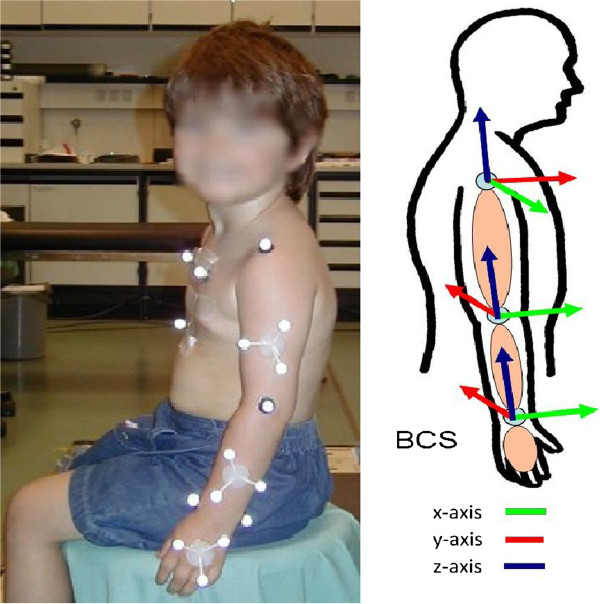
**Left: Marker setup for motion analysis of the upper extremity.** Right: Anatomical coordinate systems of the upper extremity.

The Vicon 3D motion analysis system records trajectories of passive reflective markers using infra-red cameras. Based on biomechanical models the markers are placed on the limbs of the subject, shown in Figure 1 on the left side [6,14]. The rigid body model of the upper extremity contains nine rigid segments: thorax, the clavicles, the upper arms, forearms and hands, as well as seven joints: the sternoclavicular joint, humerothoracic joints, elbow joints and wrist joints. Segments are spatially defined by a minimum of three non-collinear markers rigidly mounted on a frame to limit inter-marker movement. Joint markers are used only for calculation of the joint centers during static calibration measurements. They are removed afterwards to reduce errors resulting from the movement of underlying bony structures [7,14].

The possibility is thus offered to determine the characteristic movement strategies of a healthy person in comparison to patients using coping movements. Due coordinate systems are transformed into anatomical axis of movement (body coordinate system – BCS), shown in Figure 1 on the right side, the joint angles for the complete joint chain of the arm can be calculated. Consequently the description of the later calculated net joint forces may be arranged in anatomical axis of movement too, which simplifies to describe their direction and the stress on the anatomical structures.

### Determination of external forces acting during movement

**Figure 2:**
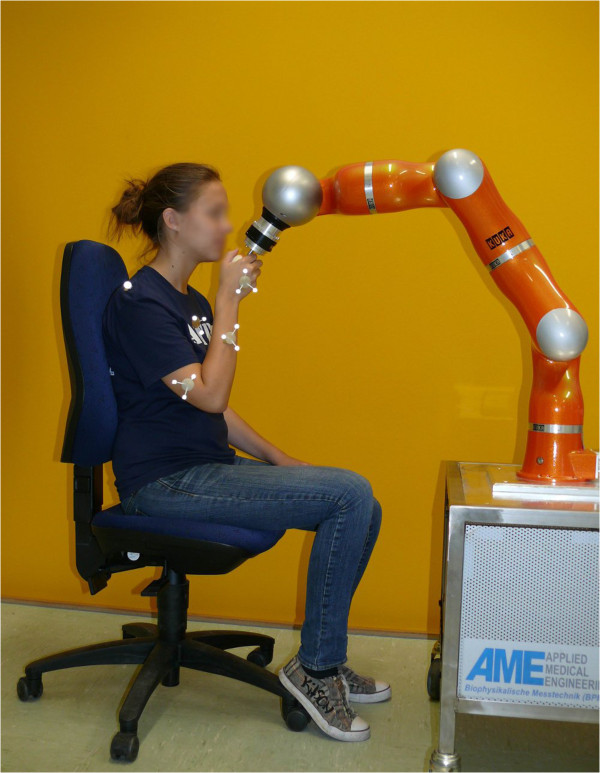
Child guided by the robot to follow the robots movement.

In order to solve the problem of standardization and to reduce the complexity of movement of the upper extremity, a six Degrees of Freedom (DoF) robot arm is used to predefine the motion. In this way movement can be tracked easily and the subject may follow the predefined curves the robot is presenting, see Figure 2. Additionally the external forces may be recorded by a six DoF force/torque sensor located at the end-effector of the robot arm. The combination of body segment parameters, motion analysis, which makes calculation of joint angles possible, and the measurement of external forces can be used for the calculation of net joint forces by means of inverse dynamics.

### Calculation of net joint forces

The first step in the inverse dynamics method is to calculate the net joint force in the most distal joint – the wrist – to later get information about the whole joint chain. For the calculation the body segment parameters of the hand as well as the external forces are required.

$${\vec{F}}_{{}_{\mathrm{wcs}\_w}}=\left(\begin{array}{l} -1\\ -1\\ 1 \end{array}\right){\vec{\cdot F}}_{{}_{\mathrm{ext}}}+m_{h}\cdot .\vec{g}-m_{h}\cdot {\vec{a}}_{{}_{h}}$$

$${\vec{F}}_{{}_{\mathrm{wcs}\_w}}$$:

Net joint force in the wrist joint

$${\vec{F}}_{{}_{\mathrm{ext}}}$$:

External forces acting on the end effector

*m*_*h*_ : Segment mass of the hand

*a*_*h*_ : Acceleration of the hand’s center of mass

$$\vec{g}$$:

Gravity

In order to show the results in anatomical axis of movement, the rotational matrix was used to transform the results from world coordinate (WCS) to body coordinate system (BCS).

$$R^{*}{}_{\mathrm{Joint}}=R_{X\_\mathrm{Joint}}\cdot R_{Y\_\mathrm{Joint}}\cdot R_{Z\_\mathrm{Joint}}$$

***R***_X _ Joint_ : Rotation matrix about x axis

$$R_{X\_\mathrm{Joint}}\left(\phi_{X}\right)=\left(\begin{array}{ccc} 1 & 0 & 0\\ 0 & \cos\phi_{X} & -\sin\phi_{X}\\ 0 & \sin\phi_{X} & \cos\phi_{X} \end{array}\right)$$

***R***_Y _ Joint_ : Rotation matrix about y axis

$$R_{Y\_\mathrm{Joint}}\left(\phi_{Y}\right)=\left(\begin{array}{ccc} \cos\phi_{Y} & 0 & -\sin\phi_{Y}\\ 0 & 1 & 0\\ \sin\phi_{Y} & 0 & \cos\phi_{Y} \end{array}\right)$$

***R***_Z _ Joint_ : Rotation matrix about z axis

$$R_{Z\_\mathrm{Joint}}\left(\phi_{Z}\right)=\left(\begin{array}{ccc} \cos\phi_{Z} & -\sin\phi_{Z} & 0\\ \sin\phi_{Z} & \cos\phi_{Z} & 0\\ 0 & 0 & 1 \end{array}\right)$$

The rotation matrix for the shoulder coordinate system is

$$R_{S}=R^{*}{}_{S}$$

The rotation of the elbow joint depends on the shoulder rotation. Additionally a rotation of 90° about the z axis is necessary to rotate shoulder to elbow joint coordinate system:

$$R_{E}=R^{*}{}_{S}\cdot R_{z90}\cdot R^{*}{}_{E}$$

The rotation matrix of the wrist joint depends on the rotation of the elbow joint:

$$R_{W}=R_{E}\cdot R^{*}{}_{W}$$

This allows the calculation of the net joint forces in body coordinate system:

$$\begin{array}{ll} {\vec{F}}_{{}_{\mathrm{Body}\_H}}= & R_{W}{}^{T}\cdot \left(\begin{array}{c} -1\\ -1\\ 1 \end{array}\right){\vec{F}}_{{}_{\mathrm{ext}}}\\ & +m_{h}\cdot \left(R_{W}{}^{T}\cdot \vec{g}\right)-m_{h}\cdot \left(R_{W}{}^{T}\cdot {\vec{a}}_{{}_{h}}\right) \end{array}$$

Analogous to the calculation of the forces in the wrist joint, forces in elbow and humerothoracic joint can be determined.

Within these transformations it is possible to calculate the net joint forces in wrist, elbow and humerothoracic joint in all three anatomical axes of movement – flexion/extension, abduction/adduction and rotational axis – to get a better understanding and use of the direction of the acting net joint forces. The net joint moments are also calculated but not further discussed in this work, because they generate a rotational movement and in absence of pathological muscular co-activation no additional translatory net joint forces.

### Simulation and measurement

The feasibility of the biomechanical inverse dynamics model is realized by simulation and later in a specific patient group of children suffering from obstetric brachial plexus palsy.

To analyze the effect of an internal rotated shoulder position on the acting net joint forces. In a first step artificial isolated flexion and extension movements were used as input for the inverse dynamics model, where the impact of different internal rotation degrees (0° to 50°) of the shoulder were investigated.

In a second step four healthy children and four children with obstetric brachial plexus palsy were instructed to perform standard movement tasks guided by the robot. For validation and comparison with the simulation, only flexion and extension movements in the shoulder are used, where movements were performed with straightened forearm and hand. The choice fell on the flexion and extension movement because it is an isolated easy to perform movement which can be guided by the robot properly. Also the flexion/extension movement in the shoulder is not directly affected by the palsy itself. It can be performed by all affected subjects with no or minor restrictions and so allows a comparison to the healthy ones. In each trial 3 repetitions of maximum shoulder flexion and extension were performed starting in full extension in a seated position in front of the robot.

## Results

**Figure 3:**
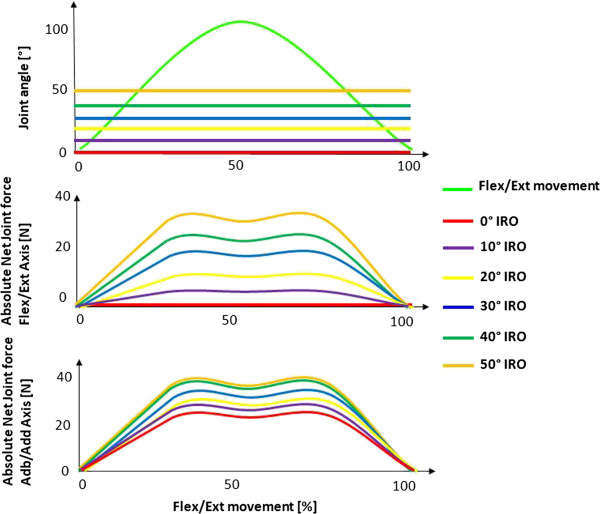
Upper: Simulation of different internal shoulder rotation positions (IRO) in shoulder flexion/extension movements Lower: Absolute net joint forces in the shoulder flexion/extension and abduction/adduction axis with internal shoulder rotation position (IRO) from 0°-50°.

**Figure 4:**
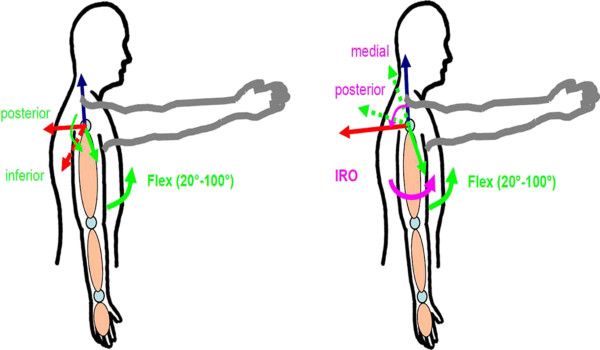
**Left: Changes of the direction of the net joint forces in anatomical flexion/extension axis from medial to posterior.** Right: Changes of the direction of the net joint forces in abduction/adduction axis from posterior to inferior.

The net joint forces acting in the anatomical flexion/extension axis of the humerothoracic joint are increased with increasing internal shoulder rotation. The joint angles of the simulation for an artificial isolated single flexion and extension movement of the humerothoracic joint with increasing internal shoulder rotation position (IRO) from 0 to 50° are shown in the upper part of Figure 3. The resulting absolute net joint forces in the anatomical flexion/extension axis as well as abduction/adduction axis of the shoulder joint during this movement with increasing internal rotation position from 0 to 50° are shown in the lower diagram of Figure 3. In Figure 4 on the left the influence of the internal rotation position of the shoulder on the direction of the net joint forces is shown. Here it can be observed that with increasing internal shoulder rotation the direction of net joint force in the anatomical abduction/adduction axis is changing from posterior (red arrow) to inferior. The stabilization function of this force in the humerothoracic joint is reduced by this change of direction. On the right of Figure 4 the change of the net joint forces in the anatomical flexion/extension axis is shown, where the direction of the net joint forces is changing from medial (green arrow) to posterior with increasing internal shoulder rotation. The sum and the increased magnitudes of these net joint forces all lead to an increased coaptation of the humerus.

**Figure 5:**
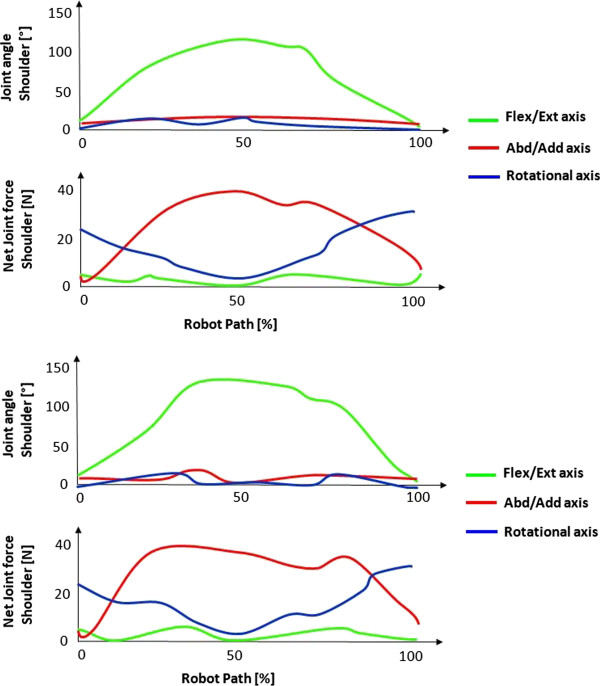
Shoulder joint angle during a full flexion/extension movement of two healthy subjects and the resulting absolute values of the net joint forces in anatomical axes.

**Figure 6:**
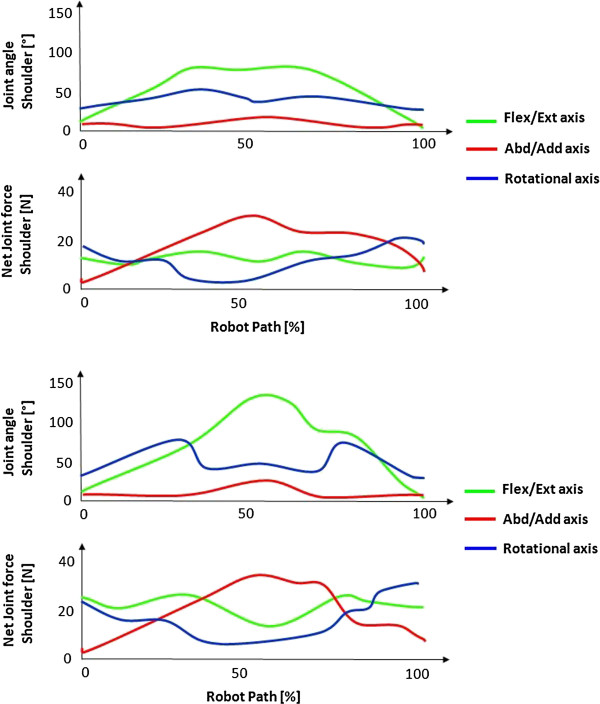
Shoulder joint angle during a full flexion/extension movement of two pathological subjects and the absolute values of the resulting net joint forces in anatomical axes.

In comparison to the simulated movement, the same increasing net joint forces in the anatomical axis of movement with increasing internal rotation position of the shoulder during flexion/extension movements can be observed in the measurements of the children suffering from obstetric brachial plexus palsy. The net joint forces during the flexion and extension movements of the shoulder with both, healthy and affected children, are shown exemplarily in two healthy subjects in Figure 5 and two affected subjects in Figure 6. In Figure 5 the movement’s path describes a movement from full extension to the subject’s maximum flexion of the shoulder and back (green curve). The blue curves represent the degree of shoulder rotation, where in Figure 5 can be observed a nearly constant value of a maximum of 10° internal shoulder rotation in the healthy subjects. The net joint forces shown in the lower diagrams show a minimal stress in the shoulder flexion/extension axis. In Figure 6 the affected children are performing the same movement but in a pathological internal shoulder rotation position of around 40°. In the lower diagrams it can be observed that there is a strong increase of the net joint force in the flexion/extension axis of the humerothoracic joint, whereas the other net joint forces are similar to the ones of the healthy children.

## Discussion

From the results of the simulated flexion and extension movement in the shoulder joint it can be observed that the net joint forces in the flexion/extension axis are increasing with increasing internal rotation position of the shoulder. Furthermore, the measurements of children with brachial plexus palsy have shown a similar behavior like the results from the simulation; again increasing net joint forces and a change of direction in the anatomical axis, flexion/extension as well as abduction/adduction, of the shoulder with increasing internal shoulder rotation can be observed.

The most common reported malformations in the shoulder joint in patients suffering from brachial plexus palsy are scapular rotation and elevation as well as the medial rotation and posterior subluxation of the humerus head [1,2,12].

To investigate the impact of the simulated and measured net joint forces in the shoulder the anatomical orientation of the humerus during shoulder flexion must be taken into account. Positive forces acting in the anatomical flexion/extension axis of the shoulder are resulting in a coaptation of the humerus moving medial-posterior. These are exactly the anatomical structures stressed by the increased net joint forces presented in simulation and measurement. The result is a growth of bone and cartilage in the humerothoracic joint in order to compensate these forces. This osseous malformation may lead to pain during movement and a reduction of the range of motion, which is also reported in literature and has been proven in image guided studies [1,2].

This presented pathological coping movement appears within subjects having a partial plexus brachialis palsy resulting in an internal rotation position of the shoulder. Patients with a full palsy often do not show this coping movement due to the full palsy both, internal and external rotators are affected. Only in absence of the antagonistic muscle group do the internal rotators shorten with no additional muscular activation and this leads to the presence of the coping movement.

The application of the inverse dynamic model based on anthropometric data combined with kinetics and kinematics appears to be a good framework for further analysis and shows a proper procedure to objectively investigate the impact of modified net joint forces on joint malformation. But there are some limitations of the model which should be mentioned and improved. The contribution of soft tissue and muscular components is not included in the biomechanical model. It is restricted to mechanically induced net joint forces, in this case coping movements resulting from muscular dysbalance. The origin of the coping movements, the muscular dysfunction, should be in investigated separately in sEMG studies. The regression presented by Zatsiorsky et. al. is based on adult values and was scaled in this paper to children’s anthropometry. For upcoming measurements there should be a step by step increase of the internal rotation of the shoulder to validate the model in an extended patient group where statistics are representative. Finally there should be investigations of patient groups with different joint malpositions and coping movements to get further insight into the impact of pathological movements and joint malposition on net joint forces.

## Conclusions

This work presents an approach to calculate net joint forces in anatomical axes of movement via a biomechanical inverse dynamics model. The simulated net joint forces show the same behavior as the results of the measurements with a patient group of children suffering from obstetric brachial plexus palsy. Concluding: Pathological coping movements resulting from neuromuscular dysfunctions have a strong impact on net joint forces during movements and are a possible reason for osseous joint malformation.

## Competing interests

All authors declare that they have no competing interests.

## Authors’ contributions

TK drafted the manuscript, worked out the simulations, acquired and processed the patient data and analyzed and interpreted the results. NP designed the biomechanical model, worked out the simulations and helped to draft the manuscript. JB designed the study and chose the patients. CDK revised the manuscript critically and supported in interpretation. All authors have read and approved the manuscript.
